# Two-Hour Tobacco Abstinence Has No Effect on Cognitive Control in Male Patients With Nicotine Dependence: An ERP Study

**DOI:** 10.3389/fpsyt.2020.604684

**Published:** 2020-12-03

**Authors:** Yanling Xue, Hongliang Zhou, Chenguang Jiang, Xiaohong Liu, Zhenhe Zhou, Jun Wang

**Affiliations:** ^1^Department of Psychiatry, The Affiliated Wuxi Mental Health Center of Nanjing Medical University, Nanjing, China; ^2^Nanjing Brain Hospital Affiliated to Nanjing Medical University, Nanjing, China

**Keywords:** nicotine dependence, event-related potential P300, P3a, P3b, tobacco abstinence

## Abstract

The average nicotine half-life in body tissues is 2 h. Understanding the influence of pure nicotine abstinence on cognitive control may be helpful in eliminating nicotine dependence (ND) and preventing smoking relapse. This study was to investigate the effects of 2-h tobacco abstinence on cognitive control in patients with ND. Twenty-five patients with ND completed event-related potential (ERP) P300 measurements at the normality state and the abstinence state. Twenty-five healthy controls (HCs) were measured with P300 twice with a 2-h time interval. HAMD and HAMA were used to assess the emotional state. Results showed that there were significant differences in Carbon monoxide (CO) levels between the abstinence state and the normality state in the ND group. There were no significant differences in HAMD and HAMA scores for the abstinence state in the ND group or the normality state in the ND group and the HC group. For P3a, P3b amplitude, and P3a latency, the main effect for ND group was significant. For P3a, P3b amplitude, and latency, the interaction effect for group × time point was not significant, and the main effect for time point was not significant. It concluded that patients with ND present cognitive control deficits, and 2-h tobacco abstinence has no effect on cognitive control deficits in male patients with ND. Our findings may be helpful in eliminating nicotine dependence and preventing smoking relapse.

## Introduction

Tobacco smoking causes nicotine dependence (ND), which leads to over 6 million deaths worldwide per year, and the World Health Organization predicts that this number will rise to 8 million per year by 2030 (1). As a stimulant that expresses its rewarding effects through the release of dopamine and other neurotransmitters in the brain, nicotine is the primary addictive component in tobacco (2).

Cognition is the ability to perform the mental actions or processes of understanding through all kinds of cerebral cortex activities, such as thought, experience, and the senses (3, 4). Executive function is a critical neurocognitive function and can be measured with neuropsychological tests. For example, the Wisconsin Card Sorting Test is a neuropsychological test of the ability to exhibit flexibility in the face of changing schedules of reinforcement (5, 6). Cognitive control belongs to an important executive function. The event-related potential (ERP) is a technique that can provide an analysis of neural activity with high temporal resolution. Moreover, it is used to measure behavioral alterations in schizophrenia, affective disorders, and addiction (7–9). To assess cognitive function at a deeper level, the ERP P300, which is a positive deflection of electric potential generated ~300–500 ms after an infrequent stimulus related to a specific event, may provide the possible neural correlates of cognitive processing.

Cognitive impairments (especially cognitive control deficits) are related to the maintenance of nicotine dependence, nicotine abstinence and the target of pharmacotherapy (10). Many previous studies have reported that patients with nicotine dependence (ND) display cognitive dysfunctions (11, 12), especially executive dysfunctions related to nicotine dependence and craving. For example, a previous study investigated the impact of executive functions, including updating, inhibition and shifting processes, on nicotine dependence and craving; the results indicated a prefrontal cortex (PFC) dysfunction affecting the inhibitory capacities of patients with ND (13). A study investigated the effects of smoking on PFC-mediated cognitive flexibility and subjective states in low- and high-nicotine-dependent individuals and found that the PFC-mediated cognitive effect of smoking as well as subjective reports vary according to the degree of nicotine dependence; smoking selectively impairs cognitive flexibility in high-nicotine-dependent individuals (14). Another study employed a reinforcement-learning task to examine the effects of smoking status on monitoring errors and conflict and found that monitoring errors and conflict are influenced by smoking status (15).

The P3 family of ERP components is a marker of cognitive control processes (16). The P300, which is evoked by family of ERP a three-stimulus oddball task, includes P3a and P3b. P3a is evoked by novel stimuli and considered a psychophysiological index of the orienting response, namely, it reflects involuntary attentional switching and attentional reorienting (17, 18). P3b represents correct responses to correct responses to target tones and P3b is associated with the identification of task-relevant target stimuli (19). There were different findings for the effect of acute nicotine on P300 (P3a or P3b) in healthy non-smokers. For example, previous studies showed that acute nicotine did not alter the P3a amplitudes and latencies in healthy non-smokers (20–22); however, a study reported that acute nicotine attenuated P3a amplitudes (23). Studies which involved the influence of nicotine on P300 (P3a or P3b) in chronic smokers indicated that the decreased P300 amplitude was associated with cigarette smoking (24, 25).

Studies using P300 also indicated that patients with ND present cognitive control deficits. A recent study investigated how the age at tobacco smoking onset affects neurophysiological measures of smoking cue reactivity and reported craving in adult smokers using an oddball paradigm P300 (26). The findings revealed that P300 amplitudes at the Cz electrode site were greater in early-onset nicotine-dependent individuals and associated with greater craving at baseline. A previous study explored the effects of nicotine deprivation (12-h nicotine deprivation) on P3a and P3b amplitudes and examined self-reported trait cognitive control as a moderator of nicotine deprivation-induced reductions in P3a and P3b amplitudes (19). This research finding showed that nicotine deprivation reduced P3b amplitude during a three-stimulus oddball task independent of trait cognitive control. However, nicotine deprivation reduced P3a only in subjects who scored lower on measures of trait cognitive control. Another study using an oddball paradigm reported that chronic nicotine-dependent patients present reduced P300 amplitudes compared to individuals who never smoked or those who had terminated smoking (27). Additionally, a previous study indicated persistent P300 amplitude reduction in nicotine-dependent patients using an auditory oddball paradigm (25).

Many studies indicated that nicotine abstinence causes physiological, psychological and cognitive symptoms (28–31). Furthermore, nicotine abstinence may produce depressive symptoms or precipitate a major depressive episode (32). Whether nicotine abstinence may impair cognition or not has been debated. Many studies have indicated that nicotine abstinence exhibits impairments in working memory during smoking abstinence in patients with ND (33–36). However, previous studies reported that smoking abstinence attenuates attentional bias toward positive stimuli (37, 38).

The psychological symptoms produced by nicotine abstinence, such as anxiety and depression, can also lead to cognitive dysfunctions. Nicotine abstinence is associated with cognitive control deficits, and these cognitive control deficits are a hallmark of nicotine abstinence that could be targeted for success in quitting smoking (10). Thus, the early withdrawal period is a vulnerable time for patients with ND and represents a critical window in which to appraise the outcome of refraining from smoking. A better understanding of the influence of pure nicotine abstinence on cognitive control function may be helpful to improve smoking cessation programmes.

Because nicotine entry into circulation is through the pulmonary system, tobacco smoking is a highly addictive form of systemic drug administration. Studies have confirmed that nicotine can reach the brain in 10–20 s, and brain nicotine concentrations increase after smoking each cigarette and then decline over 20–30 min as nicotine redistributes to other organs or tissues; the average nicotine half-life in body tissues is 2 h (39). Previous studies that investigated nicotine abstinence on cognitive control function focused on nicotine deprivation for more than 12 h (11, 19, 31, 40, 41) but could not avoid confounding the cognitive control impairment induced by pure nicotine abstinence and the psychological symptoms produced by nicotine abstinence.

In summary, cognitive control deficits, which can be measured with neuropsychological tests, are related to the maintenance of nicotine dependence, nicotine abstinence and the target of pharmacotherapy. An ERP P300 study may provide a more definitive answer regarding the effects of nicotine deprivation on P3a- or P3b-related neural activity. Additionally, nicotine withdrawal-induced cognitive performance deficits are typically not discovered within 2 h of tobacco deprivation (42, 43). Understanding the influence of pure nicotine abstinence on cognitive control function may be helpful in eliminating nicotine dependence and preventing smoking relapse. However, to date, the influence of pure nicotine abstinence on cognitive control function is still unclear.

In this study, male patients with ND were selected as subjects. The cognitive control deficits of nicotine deprivation were measured with P300, including P3a and P3b components, which is evoked by a three-stimulus oddball task. To guarantee the influence of pure nicotine abstinence on cognitive control function, cognitive performances were collected at baseline and after 2 h of tobacco deprivation. The hypothesis of this study is that male patients with ND present abnormal P300 components and 2-h tobacco abstinence has no effect on cognitive control deficits. The purpose of this study was to investigate the effects of 2-h tobacco abstinence on cognitive control deficits in patients with ND.

## Method

### Time and Setting

This study was conducted in the Department of Psychiatry, The Affiliated Wuxi Mental Health Center of Nanjing Medical University, Wuxi, People's Republic of China, from January 01, 2018, to March 31, 2020.

### Diagnostic Approaches and Participants

This study included patients in the ND group and a healthy control (HC) group. The criteria for inclusion in the ND group included (a) meeting the Diagnostic and Statistical Manual of Mental Disorders, Fifth edition (DSM-5) criteria for current nicotine dependence; (b) age range from 18 to 60 years old; (c) had not previously quit smoking and reported smoking more than 10 cigarettes per day in the last 6 months; (d) no smoking cessation in the past 12 months; and (e) no neurological illness or psychiatric disorders as determined by clinical evaluations and medical records or alcohol/other substance dependence. The criteria for inclusion in the HC group included (a) not meeting the criteria for any DSM-5 axis I disorder or personality disorders, as assessed by the Structured Clinical Interview for DSM-5 (SCID-5, Chinese version); (b) age range from 18 to 60 years old; (c) no history of any kind of mental disorder; and (d) no physical illness.

In the present study, 25 patients with nicotine dependence were recruited as the ND group. Patients with ND were recruited from the Smoking Cessation Clinic of Psychiatry Department, The Affiliated Wuxi Mental Health Center of Nanjing Medical University, Wuxi, People's Republic of China. Patients with ND only smoked cigarettes, not electronic or other tobacco products. Twenty-five healthy persons were recruited as the HC group. HCs were recruited from a group of citizens who lived in Wuxi City, China, through local advertisements. All of the participants were Chinese.

### Experimental Procedures

All participants were prohibited from drinking any soft drinks, such as coffee, tea, or other recreational drugs, at least 12 h prior to the experiment. That was confirmed verbally before the test day. On the day of the ERP recording, two psychiatric resident physicians collected patient medication information, demographic data, and clinical characteristics and confirmed/excluded a diagnosis of current nicotine dependence. The Annett handedness scale was used for the assessment of handedness (44). Nicotine dependence levels were measured with the Fagerstrom Test of nicotine dependence (FTND) (45). Both patients with ND and HCs were hearing evaluated previous to inclusion in study, and all participants' hearings were in the normal level.

Before starting the experiment, all participants were instructed to try their best to complete the task as quickly and accurately as possible. The authenticity of the 2-h nicotine abstinence was determined by expiratory carbon monoxide levels measured using the QT-200PLUS portable detector of carbon monoxide (CO) (Shenzhen Wellcome Technology Co., Ltd., China). The CO levels of patients with ND were measured 10 min before the experiment. The CO level in the expired air was verified as no more than 6 parts per million (ppm) during the abstinence state, which showed a distinct reduction for each patient compared to that measured during the normal smoking state (more than 10 ppm). The CO levels of HCs also were measured 10 min before the experiment, and the CO levels of all HCs in the expired air were verified as no more than 3 ppm.

All patients with ND were measured with ERP P300 at the normality state (time 1, i.e., just after the last cigarette smoked) and abstinence (time 2, i.e., just at 2 h after the last cigarette smoked). During the 2 h abstinence phase, all participants were arranged to stay in a comfortable room, and all participants skimmed through a newspaper or sat in the room peacefully depending on their own desire. To avoid the practice effect, the HCs were measured with ERP twice with a 2-h time interval (corresponding to time 1 and time 2). The anxiety and depression of all participants were assessed with the Hamilton Anxiety Scale (HAMA) and Hamilton Depression Scale (HAMD, 17-item edition).

All experimental procedures were approved by the Ethics Committee on Human Studies, the Affiliated Wuxi Mental Health Center of Nanjing Medical University, Wuxi, China, and were conducted in accordance with the Declaration of Helsinki. All participants provided written informed consent to participate, and all participants were compensated 300.00 Chinese Yuan (CNY) plus travel costs.

### Event-Related Potential Measurement

The event-related potential measurement was obtained from a recent study (46). The BioSemi Active Two system (BioSemi Inc., Amsterdam, Netherlands) was employed for the continuous electroencephalogram recording. The digitization rate was 512 Hz; the bandpass was DC-104 Hertz (Hz), and a common mode sensor served as the reference (PO2 site) using a 64-channel electrode cap. Electrooculogram electrodes were placed below and at the outer canthi of the left eye. A three-stimulus (novelty) auditory oddball paradigm was employed to evoke ERP P3a and P3b. There were 400 binaural, 80 decibel (dB) tones of 50 millisecond (ms) duration stimuli presented to the participants through foam insert earphones. Twelve percent of the stimuli were target tones (1,500 Hz), 12% were infrequent “novel” sounds (a bird call or a water drop), and 76% were standard tones (1,000 Hz), with an inter-stimulus interval varying between 1.8 and 2.2 s. Stimuli presentation was randomized. The electrical impedance was monitored. The duration of the whole P300 paradigm is 8 min. Participants were in a sound attenuated chamber. All subjects were instructed to press the computer mouse in response to target tones only. Clicking that occurred between 100 and 900 ms after the tone was confirmed as a correct response. Before the formal trial, there was a practice block to make sure participants understood the task.

### Event-Related Potential Data Analysis

ERP data were analyzed using BrainVision Analyzer 2.0 (Brain Products GmbH, Munich, Germany). According to a previous study, P3a was analyzed at the Cz electrode site because it is the largest in the frontal regions, and P3b was analyzed at the Pz electrode site because it is the largest in the parietal regions (46–48). An average of the mastoids was reference and bandpass filtered between 0.01 and 20 Hz using a zero phase shift Butterworth filter. Data were segmented by stimulus marker from −100 to 1000 ms, responses to novel sounds (a bird call or a water drop) were employed for P3a, and correct responses to target tones were employed for P3b. Segments were baseline corrected using −100–0 ms pre-stimulus time and eye-blink corrected using established measures (46). Artifact rejection for individual channels was performed, and a given segment was rejected if the voltage gradient exceeded 50 μV/ms, the amplitude was ±100 μV, or the signal was flat (<0.5 μV for more than 100 ms). Segments were averaged across stimulus markers, the P3a amplitude peak was chosen to be 250–450 ms, and the P3b amplitude peak was chosen to be 280–650 ms.

### Data Analysis

Statistical Program for Social Sciences software version 19.0 (SPSS, IBM Corporation, Armonk, NY, USA) was employed for the data analysis. Mean age and education were compared between the ND group and the HC group using two-tailed *t* tests, and handedness was compared using the Pearson chi-square test. CO levels were compared in the ND group using paired-samples *t* tests. HAMD scores, HAMA scores, behavioral data [i.e., reaction time (RT), rate of correct responses (Hit rate), and rate of incorrect responses (Error rate) for target stimuli] and the mean amplitudes and the mean latencies of P3a and P3b were compared between the ND group and the HC group using a 2 (ND group vs. HC group) × 2 (time 1 vs. time 2) repeated-measures analysis of variance (ANOVA). The degrees of freedom of the *F* ratio were corrected according to the Greenhouse–Geisser method. Least square difference tests were performed as *post-hoc* analyses if indicated. Correlation analysis between HAMD scores, HAMA scores and measures of P3a and P3b in ND and HC groups separately at time 1 and time 2 were conducted by Pearson's *r*. Alpha values of 0.05 were considered significant.

## Results

### Demographic Characteristics of Participants

The demographic characteristics of all participants are shown in Table 1. There were no significant differences in mean age, mean education years, or handedness between the ND group and the HC group.

**Table 1:** Demographic data of participants.

**Variable**	**ND (*n* = 25)**	**HC (*n* = 25)**	**Test statistic**
Mean age (SD)	32.0 (10.3)	30.9 (6.9)	*t* = 0.421, *p* = 0.676
Age range	22–59	23–58	–
Education (SD)	14.2 (2.3)	15.0 (2.1)	*t* = −0.150, *p* = 0.256
FTND (SD)	7.5 (1.3)	–	–
Handedness (R/M/L)	9/7/9	10/7/8	χ^2^ = 0.176, *p* = 0.890
The number of cigarettes smoked per day (SD)	17.5 (1.5)	–	–

*ND, Nicotine dependence; HC, Healthy control; SD, Standard deviation; R, Right; M, Mixed; L, Left; FTND, Fagerstrom Test of Nicotine Dependence*.

### Comparisons of CO Levels in the ND Group

Based on the paired-samples *t* test, there were significant differences in CO levels between the abstinence state (mean 5.6 ppm; SD: 1.2) and the normality state (mean 12.1 ppm; SD: 1.0) in the ND group (*t* = 3.465, *p* = 0.000), and CO levels in the abstinence state were less than that in the normality state.

### Comparisons of HAMD and HAMA Between the ND Group and the HC Group

As shown in Table 2, using HAMD and HAMA scores as dependent variables, a 2 × 2 repeated measures ANOVA with group (ND group vs. HC group) as a between-subjects factor and time point (time 1 vs. time 2) as a within-subjects factor revealed that the interaction effect for group × time point was not significant (for HAMD, *F*_*1, 48*_= 1.894, *p* = 0.162; for HAMA, *F*_*1, 48*_ = 1.934, *p* = 0.178;); the main effect for group and time point was not significant (for HAMD, *F*_*1, 48*_= 195.320,187.113, *p* = 0.235, 0.265; for HAMA, *F*_*1, 48*_= 180.240, 179.089, *p* = 0.188, 0.197).

**Table 2:** HAMD scores, HAMA scores and behavioral data [mean (SD)] in the ND group (*n* = 25) and HC group (*n* = 25).

**Variable**	**ND**					**HC**				
	**HAMD**	**HAMA**	**RT (ms)**	**Hit rate**	**Error rate**	**HAMD**	**HAMA**	**RT (ms)**	**Hit rate**	**Error rate**
Time 1	13.8 (2.5)	10.3 (2.4)	395.1 (25.2)	0.825 (0.040)	0.208 (0.050)	13.4 (1.9)	10.5 (2.0)	382.1 (19.8)	0.915 (0.038)	0.102 (0.0113)
Time 2	13.6 (2.2)	10.1 (2.8)	394.3 (22.0)	0.826 (0.397)	0.207 (0.488)	13.3 (2.1)	10.2 (2.6)	385.4 (20.3)	0.920 (0.029)	0.101 (0.012)

*ND, Nicotine dependence; HC, Healthy control; SD, Standard deviation; RT, reaction time; Hit rate, rate of correct responses for target stimuli; Error rate, rate of incorrect responses for target stimuli*.

### Behavioral Data Analysis

As shown in Table 2, using RT, Hit rate and Error rate as dependent variables, a 2 × 2 repeated measures ANOVA with group (ND group vs. HC group) as a between-subjects factor and time point (time 1 vs. time 2) as a within-subjects factor revealed that the interaction effect for group × time point was not significant (for RT, *F*_*1, 48*_ = 2.043, *p* = 0.177; for Hit rate, *F*_*1, 48*_ = 1.986, *p* = 0.180; for Error rate, *F*_*1, 48*_ = 2.123, *p* = 0.216), and the main effect for time point was not significant (for RT, *F*_*1, 48*_ = 0.749, *p* = 0.270; for Hit rate, *F*_*1, 48*_ = 0.912, *p* = 0.294; for Error rate, *F*_*1, 48*_ = 0.883, *p* = 0.293); however, the main effect for group was significant (for RT, *F*_*1, 48*_ = 7.840, *p* = 0.027; for Hit rate, *F*_*1, 48*_ = 6.542, *p* = 0.019; for Error rate, *F*_*1, 48*_ = 6.380, *p* = 0.021). RT in ND group were longer than that in HC group; Hit rate in ND group were lower than that in HC group; Error rate in ND group were higher than that in HC group.

### ERP Data Analysis

All ERP data are shown in Table 2. Using P3a and P3b as dependent variables, a 2 × 2 repeated-measures ANOVA was performed on mean amplitudes and mean latencies, respectively, with the group (ND group vs. HC group) as the between-subjects factor and time point (time 1 vs. time 2) as the within-subjects factor.

#### P3a Component

**Figure 1:**
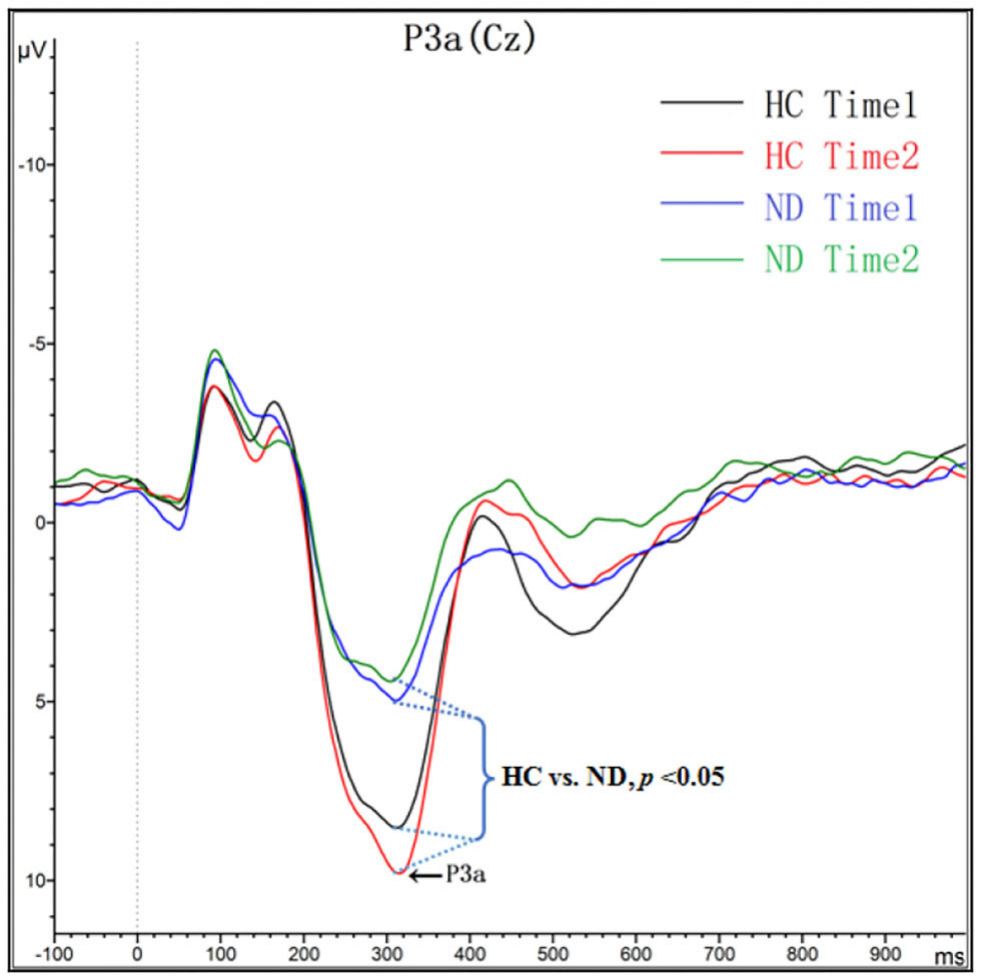
Grand averaged ERP P3a was elicited by a three-stimulus (novelty) auditory oddball paradigm for the ND group (purple lines and green lines) and the HC group (black lines and red lines) at time 1 and time 2. The P3a components were presented within a 250–450 ms latency window at the Cz electrode site.

As shown in Figure 1 and Table 3, for amplitude, the interaction effect for group × time point was not significant (*F*_*1, 48*_ = 2.147, *p* = 0.149), and the main effect for time point was not significant (*F*_*1, 48*_ = 0.262, *p* = 0.611); however, the main effect for group was significant (*F*_*1, 48*_ = 28.336, *p* = 0.000). P3a amplitudes in ND group were lower than that in HC group. For latency, the interaction effect for group × time point was not significant (*F*_*1, 48*_ = 1.942, *p* = 0.17), and the main effect for time point was not significant (*F*_*1, 48*_ = 0.089, *p* = 0.767); however, the main effect for group was significant (*F*_*1, 48*_ = 5.354, *p* = 0.025). P3a latencies in ND group were longer than that in HC group.

**Table 3:** ERP data [mean (SD)] in the ND group (*n* = 25) and HC group (*n* = 25).

**Variable**	**ND (time 1)**		**ND (time 2)**		**HC (time 1)**		**HC (time 2)**	
	**A (uV)**	**L (ms)**	**A (uV)**	**L (ms)**	**A (uV)**	**L (ms)**	**A (uV)**	**L (ms)**
P3a	5.9 (3.4)	310.8 (25.5)	5.4 (2.9)	306.2 (26.2)	10.8 (4.8)	288.6 (31.4)	11.7 (4.8)	295.7 (32.3)
P3b	4.6 (2.9)	389.5 (74.1)	4.5 (3.0)	397.7 (78.1)	6.4 (3.7)	362.6 (72.1)	6.8 (3.4)	374.2 (71.9)

*ND, Nicotine dependence; HC, Healthy control; SD, Standard deviation; A, Amplitudes; L, Latencies*.

#### P3b Component

**Figure 2:**
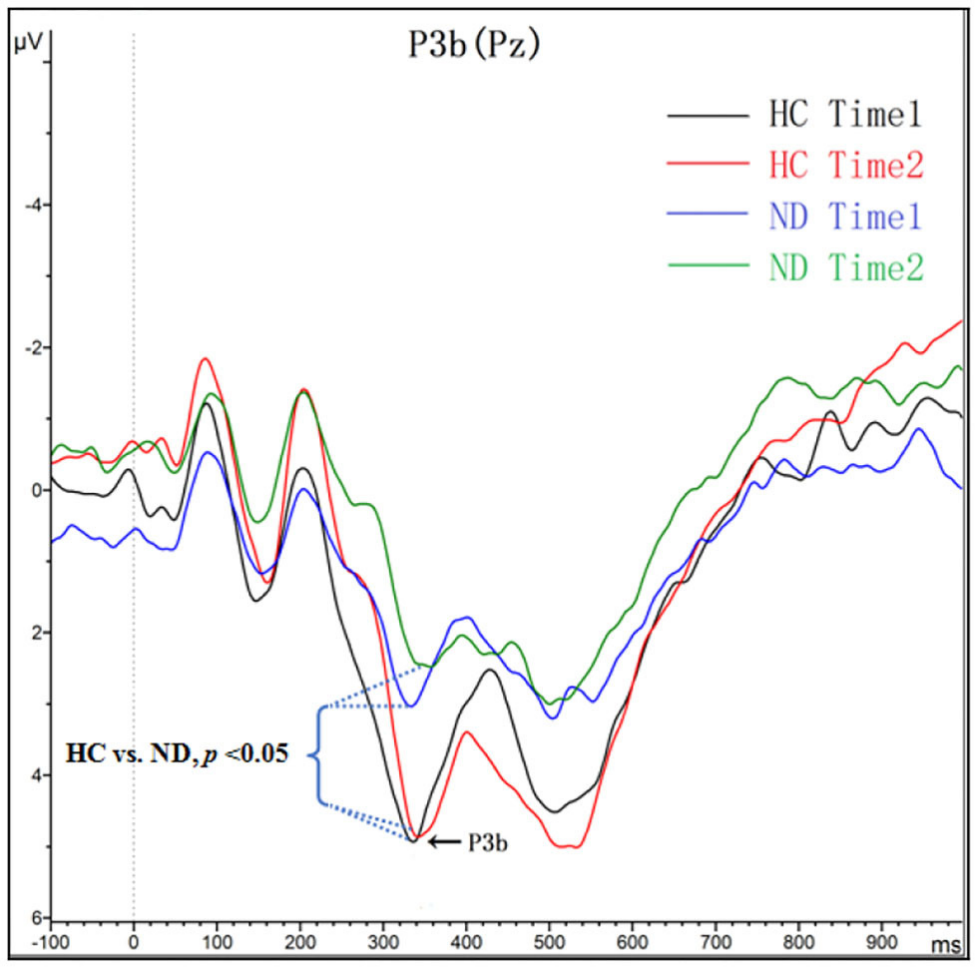
Grand averaged ERP P3b was elicited by a three-stimulus (novelty) auditory oddball paradigm for the ND group (purple lines and green lines) and the HC group (black lines and red lines) at time 1 and time 2. The P3b components were presented within a 280–650 ms latency window at the Pz electrode site.

As shown in Figure 2 and Table 3, for amplitude, the interaction effect for group × time point was not significant (*F*_*1, 48*_ = 0.277, *p* = 0.601), and the main effect for time point was not significant (*F*_*1, 48*_ = 0.121, *p* = 0.730); however, the main effect for group was significant (*F*_*1, 48*_ = 5.425, *p* = 0.024). P3a amplitudes in ND group were lower than that in HC group. For latency, the interaction effect for group × time point was not significant (*F*_*1, 48*_ = 0.043, *p* = 0.836), and the main effect for time point was not significant (*F*_*1, 48*_ = 1.432, *p* = 0.237); the main effect for group was not significant (*F*_*1, 48*_ = 1.705, *p* = 0.198).

### Correlation Analysis Between HAMD Scores, HAMA Scores, and Amplitudes and Latencies of P3a and P3b in ND and HC Group

HAMD scores were not correlated with amplitudes and latencies of P3a and P3b in ND and HC group at time 1 and time 2 (for amplitudes and latencies of P3a in ND group: *r* = 0.135, 0.236, *p* = 0.325, 0.420; for amplitudes and latencies of P3a in HC group: *r* = 0.192, 0.208, *p* = 0.294, 0.383. for amplitudes and latencies of P3b in ND group: *r* = 0.367, 0.330, *p* = 0.221, 0.329; for amplitudes and latencies of P3b in HC group: *r* = 0.204, 0.217, *p* = 0.270, 0.293.).

HAMA scores were not correlated with amplitudes and latencies of P3a and P3b in ND and HC group at time 1 and time 2 (for amplitudes and latencies of P3a in ND group: *r* = 0.241, 0.196, *p* = 0.228, 0.310; for amplitudes and latencies of P3a in HC group: *r* = 0.239, 0.211, *p* = 0.296, 0.285; for amplitudes and latencies of P3b in ND group: *r* = 0.372, 0.345, *p* = 0.193, 0.256; for amplitudes and latencies of P3b in HC group: *r* = 0.215, 0.229, *p* = 0.264, 0.280).

## Discussion

This study is the first to clarify the effects of 2-h tobacco abstinence on cognitive control deficits in male patients with ND using ERP P300 measurement, including P3a and P3b components, which is evoked by a three-stimulus oddball task. Our results showed that patients with ND elicited a reduction in P3a and P3b amplitude as well as a prolonged P3a latency, and P3a and P3b amplitudes and latencies did not change after 2-h tobacco abstinence. Additionally, patients with ND showed a prolonged RT, a reduced Hit rate as well as an increased Error rate for the target stimuli; HAMD and HAMA scores were not correlated with amplitudes and latencies of P3a and P3b in ND group at just after the last cigarette smoked and abstinence. We verified the hypothesis, i.e., male patients with ND present abnormal P300 components and 2-h tobacco abstinence has no effect on cognitive control deficits.

P3a mainly involves a broad network of cortical regions, including the prefrontal cortex, cingulate gyrus, and hippocampus (49). P3a reflects evidence that transient activation in the neural network is involved in a variety of cognitive tasks that demand continual updating of task-set information for the selection of goal-directed actions (50). Additionally, P3a reflects the initial unhitching of the focus of attention from current information with the aim of preparing to switch attention (50). Previous studies reported that acute nicotine administration may alleviate cognitive dysfunction with increased amplitudes of P3a or P3b, and these effects are relative to information processing task difficulty, amount smoked and nicotine level (51–54). Consistent with previous studies (19, 25), our results showed a reduction in P3a amplitude and a prolonged P3a latency in the patient group, which deduces that nicotine dependence might lead to cognitive control dysfunctions, i.e., the dysfunction of an involuntary switch of attention.

P3b represents correct responses to target tones, and P3b is associated with the identification of task-relevant target stimuli (19). It has been confirmed that P3b is both a trait and state biomarker (46). Because this study was a cross-sectional study, we found a reduction in P3b amplitude compared with normal controls, which may not indicates that cognitive control deficits in patients with ND are trait dependent or state dependent.

According to a previous study, the average nicotine half-life in body tissues is 2 h (39). Our results showed no occurrence of withdrawal symptoms, such as anxiety and depression. More than 12 h of nicotine abstinence may produce many psychological symptoms. Many studies have proven that psychological symptoms, such as anxiety and depression, can lead to cognitive impairments. Previous studies have focused on nicotine abstinence for more than 12 h (11, 19, 31, 40, 41) and found a reduction in both P3a and P3b amplitudes, which indicated that 12-h nicotine abstinence may cause neurocognitive impairments (19). However, in this study, ERP P300 was measured after 2 h of tobacco abstinence, which prevented cognitive status from being induced by psychological symptoms. Our results showed that, compared with the normal smoking state, 2-h tobacco abstinence did not improve or deteriorate P3a and P3b amplitude and latency in patients with ND, which indicates that 2-h tobacco abstinence has no effect on cognitive control deficits.

In conclusion, patients with ND present cognitive control deficits, and after 2 h of tobacco deprivation, the cognitive control deficits do not improve. Specifically, 2-h tobacco abstinence has no effect on cognitive control deficits in male patients with ND. The implication of the findings is that understanding the influence of pure nicotine abstinence on cognitive control may contribute new insights into the neural mechanism of nicotine abstinence in male patients with ND. Furthermore, our results may be helpful in focusing on therapeutic target for eliminating nicotine dependence and preventing smoking relapse.

There are some limitations of this study. First, the results must be considered preliminary due to the small sample size. Future studies with larger sample sizes and the same ERP parameters are needed to confirm the results of this study. Second, in this study, no measures of blood cotinine levels were used to determine the primary metabolite of nicotine precisely. Third, no withdrawal questionnaire was used to assess the withdrawal level for ND patients. Fourth, this study excludes female smokers, hence its applicability to the general population may be limited, further examination of this effect on female smokers is necessary in the future study. Finally, because of the deficient spatial resolution of ERPs, further studies with functional magnetic resonance imaging (fMRI), positron emission tomography (PET) or magnetoencephalography (MEG) should be conducted to determine the influence of 2-h nicotine abstinence on cognitive control deficits.

## Data Availability Statement

The raw data supporting the conclusions of this article will be made available by the authors, without undue reservation.

## Ethics Statement

The studies involving human participants were reviewed and approved by the Ethics Committee on Human Studies, the Affiliated Wuxi Mental Health Center of Nanjing Medical University, Wuxi, China. The patients/participants provided their written informed consent to participate in this study.

## Author Contributions

YX, HZ, JW, and ZZ designed the study. YX, HZ, CJ, XL, JW, and ZZ contributed to the acquisition of the data, analyzed the data, interpreted the results, and drafted the manuscript. ZZ wrote the paper. All the authors critically reviewed the content and approved the final version for publication.

## Conflict of Interest

The authors declare that the research was conducted in the absence of any commercial or financial relationships that could be construed as a potential conflict of interest.
